# Perinatal antibiotic exposure alters composition of murine gut microbiota and may influence later responses to peanut antigen

**DOI:** 10.1186/s13223-018-0263-8

**Published:** 2018-11-01

**Authors:** Rozlyn C. T. Boutin, Zach Dwyer, Kyle Farmer, Chris Rudyk, Mark R. Forbes, Shawn Hayley

**Affiliations:** 1 Department of Neuroscience, Carleton University, 1125 Colonel By Drive, Ottawa, K1S 5B6 ON Canada; 2 Department of Biology, Carleton University, 1125 Colonel By Drive, Ottawa, K1S 5B6 ON Canada

**Keywords:** Allergic, Cytokine, TNF-α, Adjuvant, Microbiota, Proteobacteria

## Abstract

**Background:**

Accumulating evidence suggests that the gut microbiota shapes developmental processes within the immune system. Early life antibiotic use is one factor which may contribute to immune dysfunction and the recent surge in allergies by virtue of its effects on gut microbiota.

**Objective and methods:**

As a first step towards determining whether a relationship exists between perinatal antibiotic induced changes in the gut microbiota and the later development of a peanut allergy, we exposed newborn mice to either the broad-spectrum antibiotic vancomycin or to a vehicle for 6 weeks and then used a novel murine model of peanut allergy.

**Results:**

Early-life treatment with vancomycin resulted in a significant shift in the gut microbiota community characterized by a reduction in the abundance of firmicutes and preponderance of inflammatory proteobacteria. Mice with an antibiotic-altered microbiota, showed a localized allergic-like response characterized by ear swelling and scratching following intra-dermal peanut antigen challenge. Likewise, circulating IgE levels were increased in antibiotic-treated mice, but no evidence of a systemic allergic or anaphylactic-like response was observed. Importantly, we utilized the naturally occurring pro-inflammatory cytokine, tumor necrosis factor-α (TNF-α), rather than the more commonly used cholera toxin, as an adjuvant together with the peanut antigen.

**Conclusion:**

Our data suggest that early antibiotic exposure promotes a shift in the gut microbiota community that may in turn, influence how mice later respond to a TNF-α + antigen challenge. However, further studies verifying the capacity of microbiota restoration to protect against allergic responses will be needed to confirm a causal role of antibiotic-induced microbiota variations in promoting allergic disease phenotypes.

## Background

Over the past few decades, the prevalence of allergic diseases such as asthma, eczema, rhinitis, and food allergy has increased at such an alarming rate that these diseases now represent some of the most common chronic childhood health conditions in affluent countries [1, 2]. Rising food allergy rates represent an especially urgent health care concern, as food allergies are often comorbid with other forms of allergy, can be life-threatening, and have only food avoidance as their primary treatment strategy [3]. The geographical localization and shear speed at which food allergy rates have risen in “Westernized” countries suggest a possible role for environmental factors in driving these trends [2]. Recent studies highlighting the importance of the community of commensal and symbiotic bacteria inhabiting the gastrointestinal tract, known as the gut microbiota, in human health suggest that the over-prescription of antibiotics to young children may represent one such factor. This practice, in combination with other factors related to antimicrobial and hygienic vigilance, may be inadvertently altering the establishment, composition, and community structure of the gut microbiota of the infant intestinal tract when the immune system is still maturing. Resulting states of gut microbiota dysbiosis may thus in turn be influencing the incidence of allergic conditions at the population level [4].

Dysbiosis-induced immunopathology and dysregulation of the infant immune system is a central tenet of the “microbiota hypothesis” [5–11], a modern variation of Strachan’s original 1989 “hygiene hypothesis”. In addition to aiding in digestion, nutrient absorption, vitamin production, and the prevention of colonization by pathogenic bacteria [12, 13], the gut microbiota plays a central role in immunity and is critical for the proper development of tolerance-inducing regulatory T (Treg) cells and immunoglobulin-producing B cells [14–18]. In light of these findings, epidemiological studies supporting the microbiota hypothesis have begun to identify links between early life factors that can alter the normal gut microbiota, including antibiotic use, and the promotion of later allergy development [19]. Antibiotics such as vancomycin are capable of inducing potent and rapid alterations to the composition and diversity of the microbial species in the intestinal tract, and trends in antibiotic prescription rates parallel those of allergic conditions [2, 6]. Antibiotic exposure early in life during the critical perinatal microbial colonization period may be of particular importance, as it appears as though there is a ‘critical window’ during this period in which the gut biota has the greatest potential to exert immunomodulatory effects on the host [17].

We experimentally tested the hypothesis that early life (perinatal) exposure to the antibiotic vancomycin would provoke a shift in the gut microbiota community that would in turn be associated with increased sensitivity to the development of an allergic response to peanut antigen. Vancomycin has previously been shown to induce strong shifts in the gut microbiota linked to asthma exacerbation [19] and is often used for difficult to treat bacterial infections, such as those caused by *Clostridium difficile*, which can result in intestinal inflammation and changes in gut microbiota [20]. Vancomycin is also often used to treat severe and life-threatening infections (including osteomyelitis, endocarditis, meningitis) caused by organisms such as methicillin-resistant *Staphylococcus aureus* (MRSA), coagulase negative *Staphylococcus*, and *Enterococcus*, as well as infections seeded from a central line, dialysis/other shunt, or prosthetic heart valve.

Our secondary hypothesis was that the pro-inflammatory cytokine, tumor necrosis factor-α (TNF-α), would act as an adjuvant to increase sensitivity to the peanut antigen when administered concurrently. While most murine allergy models involved the use of cholera toxin (CT) as an adjuvant [21, 22], we opted for TNF-α due to its lower non-specific toxicity and the fact that as an endogenous cytokine, with well-known inflammatory consequences. We previously found that a single injection of TNF- α sensitized mice to the behavioural and hormonal consequences of later exposure to a bovine serum albumin (BSA) antigen 3–4 weeks later [23, 24].

## Methods

### Animals

Female and male C57BL/6 mice were purchased from Charles River Laboratories at 6–8 weeks of age. At the time of breeding, female breeders were 17–19 week-old second-time breeders, and males were first-time breeders 6–8 weeks old. Male mice were bred in-house and only male offspring were used in all experiments. Mice were housed in a standard housing environment on a 12-h light–dark cycle and received water and peanut-free Harlan Laboratories 2014 Rodent Chow food ad libitum for the duration of the experiment. All experiments were approved by the Carleton University Animal Care Committee.

### Crude peanut extract

Crude peanut extract (CPE) for the intradermal challenge was isolated from partially defatted peanut flour (12% fat, light roast) generously donated by the Golden Peanut Company (Alpharetta, GA). Briefly, a 1:8 (w:v) solution of peanut flour and 1 M NaCl adjusted to pH 9.5 using NaOH was mixed at room temperature for half an hour and then centrifuged at 4 °C and 5000×*g* for 25 min to obtain the supernatant. The supernatant was collected and the pH was adjusted to 4.0 before being centrifuged at 4 °C for 50 min at 1000×*g*. Finally, the protein isolate was obtained by washing the precipitate with water adjusted to pH 4.0. The CPE used for the intradermal challenges was made up as a 10 µg/µL solution in distilled water.

### Antibiotic treatment

Beginning on the day of parturition, six dams and their pups received a clinically relevant dose [19, 25] of vancomycin (Sigma-Aldrich), an antibiotic that targets gram-positive bacteria, through their drinking water (100 mg/L) for 3 weeks. Antibiotic water was changed every 3 days and phosphate buffer solution (PBS) was added to the water (0.5 mM) to facilitate vancomycin dissolution as per the instructions of the manufacturer. At 3 weeks of age (Day 22), pups were weaned and remained housed with their littermates while continuing to receive vancomycin in their drinking water (100 mg/L) until they were 6 weeks old (Day 42). The remaining dams and pups received normal drinking water with PBS (0.5 mM) as a control over the same time period. After Day 42, all pups received normal tap water for the remainder of the experiment.

### Murine model of peanut allergy

#### Sensitization phase

At 6 weeks of age and following the antibiotic treatment (Day 43), mice were weaned and placed into individual housing. One-third of the pups in each antibiotic/control treatment condition (i.e. 7–9 mice/group) were orally exposed to peanut antigen and TNF-α (TNF-α + PB-sensitized group). Specifically, these mice received 10 mg of peanut butter (equivalent to 2.7 mg peanut protein; Kraft All Natural peanut butter) in 100 µL distilled water spread onto 0.5 g of a digestive cookie (Mr. Christie’s Arrowroot^®^ cookies). In a pilot study (unpublished data), we found that 2 µg of TNF-α acts as an effective adjuvant to elicit an allergic reaction (ear swelling) upon subsequent exposure to the peanut protein antigen through an ear punch coated in CPE or peanut butter oil. Hence, TNF-α was given together with the peanut protein as an adjuvant to boost the immune response to the peanut antigen. Another one-third of the mice in each antibiotic/control condition were exposed to 2 µg of TNF-α alone (TNF-α group). The remaining third of the mice (control group)_ received 100 µL of distilled water alone on a digestive cookie.

#### Challenge phase

**Fig. 1:**
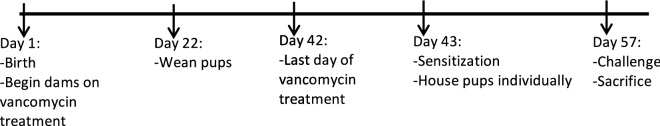
Newborn male mice reared on vancomycin or tap water from birth until 42 days of age were sensitized with TNF-α alone, TNF-α and peanut butter (PB) together or distilled water (H_2_O) on Day 43. On Day 57, all mice were challenged with an ear punch coated in crude peanut extract (CPE) and scored for subsequent ear swelling and scratching reactions. One hour later, all mice were challenged with an injection of PB and scored for signs of allergy/scratching over 1 h, after which time all mice were sacrificed. Fecal samples were taken from mice throughout the study period and analyzed for their microbial contents using Illumina sequencing

Two weeks after the sensitization treatment (Day 57), all mice were challenged intra-dermally with an ear punch. The intra-dermal ear punch challenge consisted of punching the left ear pinna of each mouse with an ear punch coated in a solution of 10 µg/mL CPE in distilled water. The right ear pinna was punched with a separate ear punch coated in distilled water as a control. Each ear punch was washed with ethanol between animals. One hour following the ear punch, all mice received an intraperitoneal (IP) injection of 5 mg of peanut butter dissolved in 100 µL of saline. We thus assessed both locally (ear) and systemically induced (IP) allergic reactions. It should be noted that IP administration is a non-physiological route of antigen delivery. Yet, this route of systemic challenge was chosen to avoid the stresses of oral gavage, as stress responses can significantly impact immune responses. One hour following the IP injection all animals were sacrificed via rapid decapitation with surgical scissors. Figure 1 shows the complete timeline of the experimental set-up.

### Allergic outcomes

#### Ear punch swelling response

At 15-min intervals for 30 min following the ear punch challenge, both the left and the right ears were scored on a scale of 0–3 for signs of swelling and irritation indicative of allergy. On this scale, 0 indicated no swelling, redness, or signs of irritation or scratching; 1 indicated an enlarged ear punch hole; 2 indicated mild swelling of the ear, and swelling at the base of the ear; and 3 indicated an enlarged ear, swelling at the base of the ear causing the ear to protrude from the head, mild redness, and/or bleeding from the ear area (Table 1). Ear sensitivity responses were calculated by subtracting the total irritation score over the 1-h period for the right ear from the total ear irritation score for the left ear. So, [Left: Experimental Ear (treated with peanut antigen)] − [Right: Control Ear (treated with vehicle)] = Ear swelling rating (corrected for non-specific swelling from punch procedure). Then, total ear swelling scores were obtained by summing the scores obtained for both 15-min time points assessed.

**Table 1:** Score, symptoms, and images for ear swelling responses observed over 1 h following an ear punch challenge

Score	Symptoms	Image
0	No signs of ear swelling (arrow), redness, or irritation	
3	Enlarged ear (arrow), swelling at the base of the ear causing the ear to protrude from the head, mild redness, and un-kept fur on the affected side of the body, and/or bleeding from the ear area	

The two extremes (scores of 0 and 3) are shown for comparison

#### Scratching and allergy/sickness responses

At the 15-min time point following the intra-dermal peanut antigen challenge, mice were observed for 2 min and assessed for scratching behaviour on a 3-point scale, with 0 indicating no scratching over the 2-min interval; 1 indicating moderate scratching over the 5-min interval (defined as 1–5 times over the 2-min interval); and 2 indicating severe or frequent scratching (defined as > 5 times over the 2-min interval) (Table 1).

Following the IP peanut butter challenge, mice were observed for 60 min for signs of allergy. Scratching behaviour was assessed at the 15, 30, 45, and 60-min time points following the IP challenge and rated on a 3-point scale, with 0 indicating no scratching over the 1 min interval; 1 indicating moderate scratching (defined as 1–2 times over the 1 min interval); and a rating of 2 used to indicate severe or frequent scratching (defined as > 2 times over the 1 min interval). Mice were also rated on sickness behaviour at these same time points using our well validated scale [24], with a score of 1 indicating normal looking; 2 indicating slight lethargy, ptosis (droopy eyes) or piloerection (puffy fur); 3 indicating very lethargic, ptosis and piloerection, curled body posture; and 4 indicating a very sick appearance, ptosis, piloerection, curled body posture, difficulty breathing, and general non-responsiveness. Total scores were obtained by summing the scores assigned at each time point assessed.

#### IgE measurements

Immediately following the 1-h observation period after the IP challenge, mice were sacrificed by rapid decapitation with surgical scissors and trunk blood was collected into chilled EDTA filled (10 µg) tubes for further analysis. Whole blood was centrifuged at 3000×*g* for 15 min to obtain plasma and stored at − 80 °C. Total circulating IgE levels were determined by sandwich ELISA using a commercial mouse IgE ELISA kit after being sent to ITR Laboratories Canada Inc (ITR SOP # IMM BIOM 42.0; available on file at ITR). Briefly, a 96-well plate was coated with a rat anti-mouse IgE monoclonal antibody. Standards and samples were then added to the wells, followed by a biotinylated rat monoclonal anti-mouse IgE detection antibody cocktail. These antibodies were subsequently detected by adding avidin-horseradish peroxidase followed by TMB Substrate Solution and absorbance was read at 450 nm with a microplate reader. The data was acquired using the Biotek Powerwave XS microplate reader coupled to Biotek Gen5 Secure software for data analysis and curve fit purposes.

### Microbial analysis

Fecal samples were taken from each of 12 cages of animals on Days 21, 28, 35, and 42 and stored at − 80 °C in a 1:1 glycerol: water solution until needed. Samples were sent on dry ice to Microbiome Insights Inc., Vancouver to be subsequently analyzed for their microbial content. Briefly, DNA was extracted from fecal samples using the Qiagen QIAamp DNA Stool Mini Kit and prepared for sequencing on a MiSeq machine using primers targeting the V3 region of the 16S rRNA gene and according to the Schloss lab protocol “16S Sequencing with the Illumina MiSeq Personal Sequencer” Standard Operating Procedure v3.1 (March 18, 2014) by Kozich et al. [25], with minor modifications. Sequences were then bioinformatically processed and analyzed following the MiSeq SOP wiki for the *mothur* v.1.34.4 software to determine the diversity and composition of the microbial contents of the samples.

### Statistics

All statistics were done using SPSS Version 20. Results were analysed using a 2 (early life treatment: antibiotic vs vehicle) × 3 (sensitization treatment: Vehicle, TNF-α, TNF-α + PB) factorial ANOVA followed by Fischer’s least significant difference (LSD) post hoc tests. For significant interactions, LSD tests were used to compare means of the effects comprising these interactions. When litter was included as a covariate in the analysis (ANCOVA) on the basis of finding significant differences in microbiome composition among litters, this did not alter findings that were significant and thus only results from the simple ANOVA are reported.

## Results

### Ear swelling scores

**Fig. 2:**
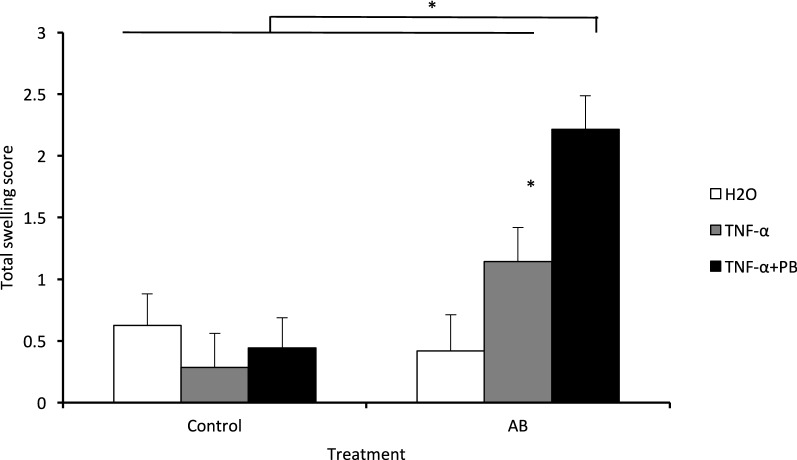
Mean total ear swelling scores of mice treated for the first 6 weeks of life with the antibiotic (AB) vancomycin are shown on the right and those that only received tap water during this time (control) are shown on the left. Following this treatment, animals were later exposed to distilled water (H2O), TNF-α, or TNF-α and peanut butter (TNF-α + PB) on Day 43. After a further 2 weeks, all mice received an ear punch challenge to the left ear with crude peanut extract in distilled water on Day 57. Ear swelling responses were scored on a scale of 0–3, with 3 being the most severe swelling response, at the 15- and 30-min time points following the ear punch challenge and were corrected for swelling responses observed in the right ear following an ear punch with distilled water received at the same time. *p < 0.05 by 2 × 3 ANOVA and Fischer’s least significant difference post hoc tests. Error bars represent one standard error

Total ear swelling responses following the ear punch challenge were significantly higher among antibiotic-treated animals as compared to the control animals (F_1,38_ = 13.356; p < 0.05; Fig. 2). Moreover, the sensitization treatment had a significant effect on ear swelling responses (F_2,38_ = 5.062, p < 0.05), and the interaction term between antibiotic treatments and sensitization treatment was significant (F_2,38_ = 6.807, p < 0.05; Fig. 2). Mean comparisons between individual groups showed that antibiotic-treated mice sensitized with TNF-α + PB treatment had significantly higher total ear scores as compared to all other groups (Fig. 2). Additionally, among mice sensitized with TNF-α alone, those previously exposed to vancomycin had significantly higher ear swelling score than those not exposed to vancomycin (p < 0.05, Fig. 2).

### Ear scratching scores

**Fig. 3:**
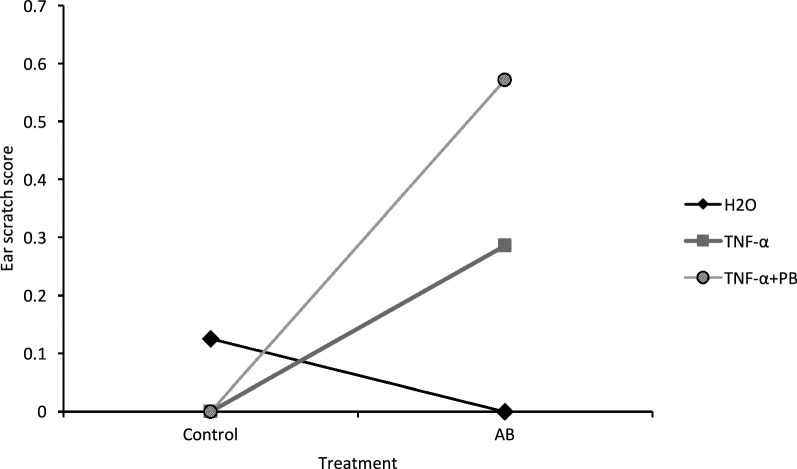
Interaction plot of mean scratching scores of mice reared for 6 weeks on the antibiotic (AB) vancomycin or on tap water (control) and then treated with either distilled water (H_2_O), TNF-α, or TNF-α and peanut butter (TNF-α + PB) (n = 6–9/group). Two week later mice were then challenged with the peanut antigen. Scratching behaviour was observed for 60 s 15 min following the ear punch challenge on a scale of 0–2, with 3 being the most severe scratching response. *p < 0.05 by 2 × 3 ANOVA and Fischer’s least significant difference post hoc tests. Antibiotic treatment significantly affected ear-scratching responses and showed a significant interaction with sensitization treatment effects (p < 0.05)

Antibiotic and control mice differed significantly in scratching behaviour (F_1,38_ = 6.098, p < 0.05), with post hoc LSD tests showing antibiotic-treated mice scratching more than control mice. There was also a significant interaction between antibiotic and sensitization treatments (F_2,38_ = 4.220, p < 0.05). Pairwise comparisons of individual means showed that scratching scores were significantly higher among vancomycin-treated mice sensitized with TNF-α + PB compared to those that received vehicle sensitization or that did not receive the antibiotic pre-treatment (Fig. 3).

### Allergy scores

**Fig. 4:**
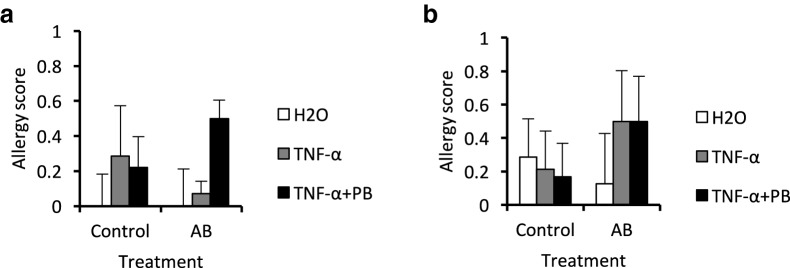
Mean allergy scores of mice reared for 6 weeks on the antibiotic vancomycin (AB) or on tap water (control) and sensitized to distilled water (H2O), TNF-α, or TNF-α and peanut butter on Day 43 at **a** 15 min and **b** 45 min on Day 57. Allergy scores out of 5 were determined at each time point based on the presence/severity of anaphylactic allergy symptoms, with a score of 5 being the most severe. Clearly, no animals approached anything near an anaphylactic reaction

Allergy scores did not significantly differ among the treatment groups. However, animals exposed to antibiotic and sensitized with TNF-a + peanut antigen appeared to exhibit slightly higher allergy scores (at 15, 30 and 45 min) than control animals (Fig. 4). However, there was substantial variability in the data, such that it appeared that there were separate populations of “responder” and “non-responder” mice and exceedingly large sample sizes would be required to complete any further analyses of such sub-groups.

### Sickness scores

No significant differences in overall sickness scores were observed among treatment groups at any time point following the IP peanut butter challenge (data not shown). Sickness ratings were generally quite low in all mice, with no animal scoring above a 2 (modest sickness symptoms) at any time point. Thus, there was no indication of an anaphylactic-like response following antigen challenge using our present experimental allergy model.

### Total plasma IgE levels

**Fig. 5:**
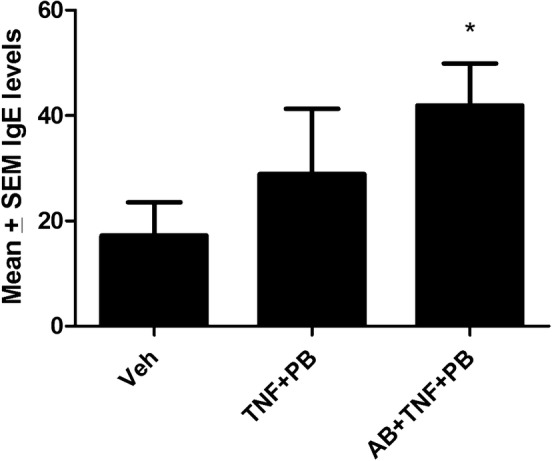
Mean circulating IgE levels in vehicle (Veh) treated controls, as well as in mice reared for 6 weeks on the antibiotic vancomycin (AB). Priming with TNF-α and peanut butter (PB) on Day 43 followed by later challenge PB again on Day 57 increased IgE levels. However, the rise only reached significance in those animals that had been pre-treated with the antibiotic, relative to controls (p < 0.05)

Paralleling the allergy scores, circulating IgE levels varied among treatment conditions but were quite variable. Indeed, although the initial ANOVA comparing treatment groups was not significant (p = 0.20), we conducted follow up comparisons based on our a priori hypothesis. These comparisons revealed that antibiotic pre-treated mice later exposed to the TNF-α + peanut antigen treatment had significantly elevated IgE levels compared to non-antibiotic vehicle treated control animals (p < 0.05). However, these antibiotic-treated mice did not differ from their non-antibiotic treated counterparts that were also sensitized with the PB + TNF-α treatment (Fig. 5).

### Microbial species

**Fig. 6:**
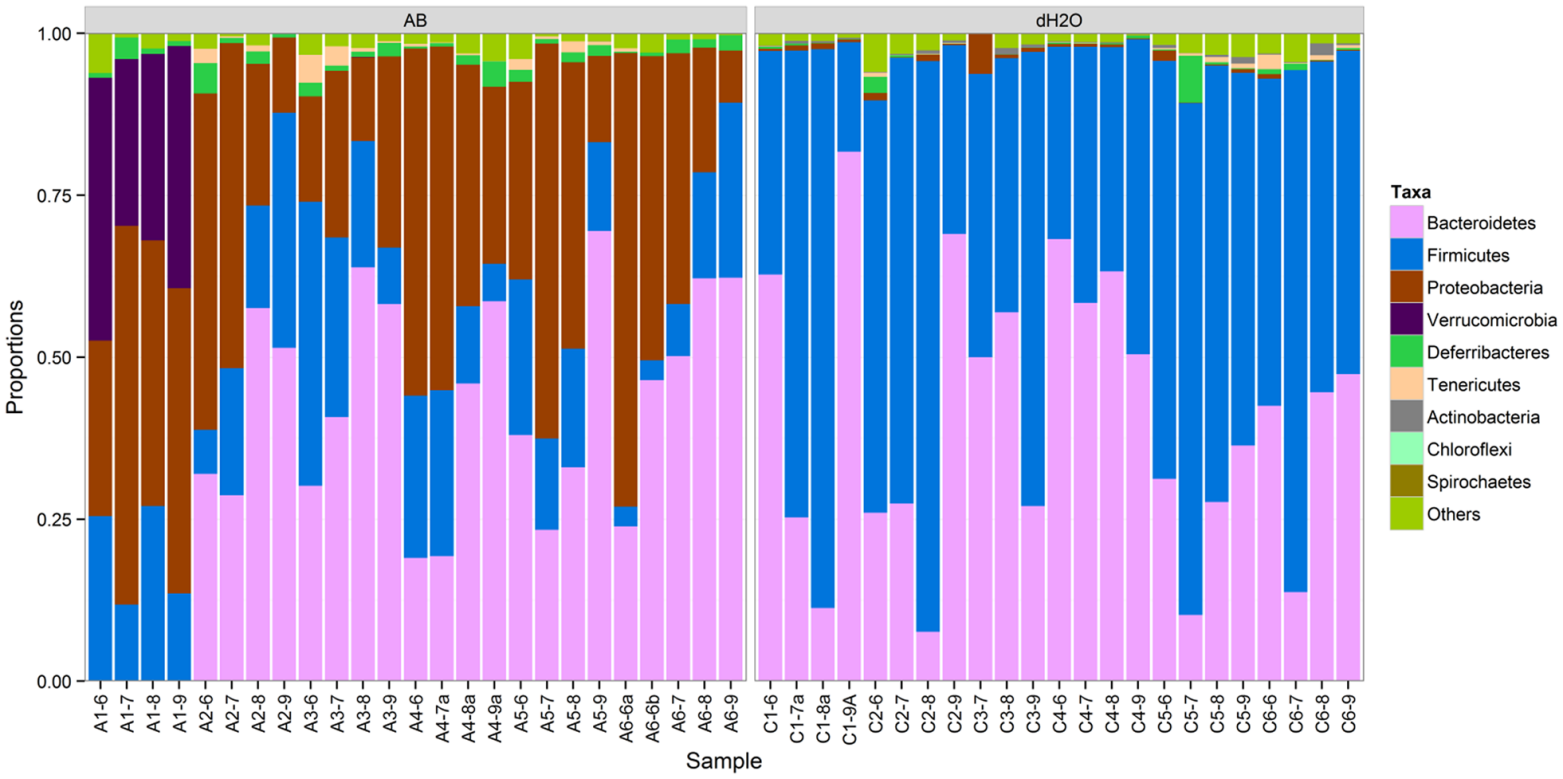
Distribution of phylum-level bacterial taxa present in the fecal samples of mice from 12 different litters reared from parturition (Day 1) for 6 weeks on the antibiotic vancomycin (AB; left side of figure) or on tap water (dH2O; right side of figure). The figure is based on operational taxonomic unit abundances aggregated into phyla. Other in the figure legend represents unclassified phyla and Acidobacteria. Litters of mice from 6 different mothers reared on antibiotics (A1-6-x) and from 6 mothers reared on regular water (C1-6-x) until weaning. Mice were housed with their mothers until weaning at Day 22 and subsequently housed with their littermates while continuing to receive either vancomycin or regular water until Day 43. Analyzed samples were taken from cages containing a single mother and all of her pups on Days 21 (A/CX-6), 28 (A/CX-7), 35 (A/CX-8) and 42 (A/CX-9)

**Fig. 7:**
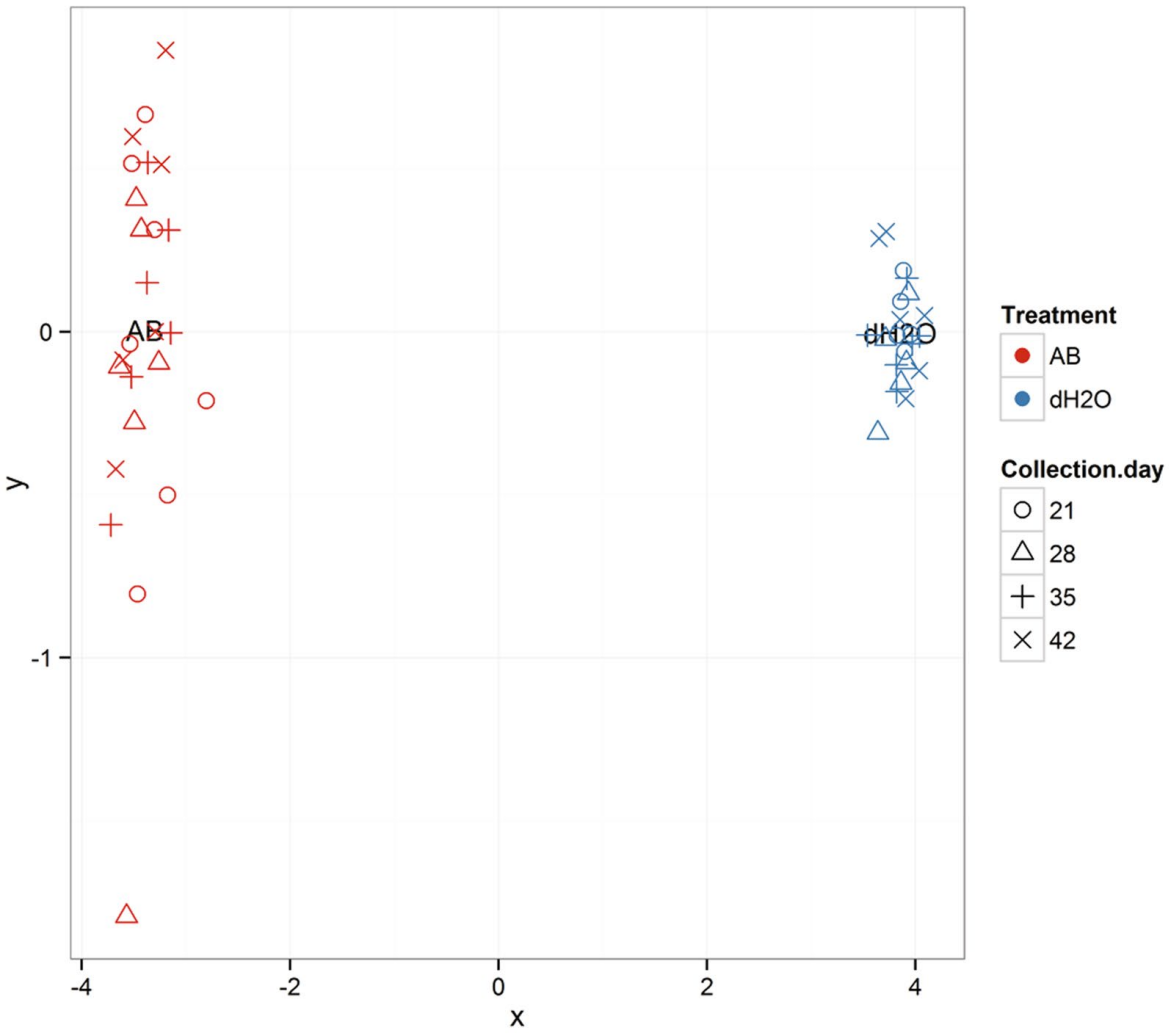
Each data point is a specific litter sampled and the different shapes represent the different sampling times. Multidimensional scaling of pair-wise Bray–Curtis distances among microbial community profiles measures how similar or different the microbial communities are, with divergence from zero reflecting greater variation as a function of time of sampling and specific litter sampled. Fecal samples taken from mice reared from parturition (Day 1) for 6 weeks on the antibiotic vancomycin (AB) or on regular water (dH2O) as controls. Litters were housed with their mothers until weaning at Day 22 and subsequently housed with their littermates until Day 43. Samples were taken on Days 21, 28, 35, and 42 from cages containing a single mother and all of her pups (Day 21) or from cages containing only pups from the same litter (Days 28, 35, and 42)

The microbial species present in the feces of antibiotic-treated and control mice differed dramatically with respect to several parameters (Fig. 6). Concomitant with an observed reduction in Firmicutes, Proteobacteria were markedly increased in relative abundance in the fecal samples of antibiotic-treated mice as compared to non-antibiotic treated mice (p < 0.05, Fig. 7). Additionally, the microbial profiles of the gut microbiota in antibiotic-treated mice exhibited greater inter-individual variability as compared to those in the non-antibiotic treated mice (Fig. 7). Litter was also found to influence the microbiome profile among antibiotic-treated mice, with the A1 litter displaying the most distinct microbial community profile as compared to animals born to dams A2–6 (Figs. 6 and 7). Importantly, in contrast to samples taken from non-antibiotic treated mice, the bacterial community composition of the fecal samples from antibiotic-treated mice showed significant changes over time (permutational analysis of variance; p < 0.05), suggesting a more reactive microbial environment.

## Discussion

Recent research in humans and rodents has suggested possible links between antibiotic use and the development of food, respiratory or skin allergies [2, 19, 26, 27]. Given that the gut microbiota is known to interact with the immune system to modulate immune cell maturation [28] and polarization, alterations in the early life gut microbiota may have an immunomodulatory influence on immune cells and responses involved in determining peanut allergy susceptibility [2, 29]. Several epidemiological studies have found associations between altered early life gut microbiota communities and an increased likelihood of later developing allergic diseases [30–36]. Moreover, advances are being made in understanding the mechanisms through which the microbiota and their microbial products alter immune cell functioning [29, 37–39]. Our present data does suggest that perinatal antibiotic alters the microbial species. This was correlated with an enhanced sensitivity to certain (ear swelling and scratching) but not other (systemic allergic and sickness scores) outcomes in response to later exposure to peanut antigen and the cytokine, TNF-α, In addition to having potential clinical relevance with regards to food allergies, these data highlight interesting interactions between immunogenic stressors over time.

Exposing newborn mice to vancomycin has been shown to have a substantive effect on the gut microbiota of these mice, altering the composition of the microbial community much more dramatically than adult exposure to vancomycin. Accordingly, we found that vancomycin exposure during the first 6 weeks of life dramatically altered the community profile of the gut microbiota of mice at the phylum level. The gut microbiota of antibiotic-treated neonatal mice had reduced levels of Firmicutes and Bacteroidetes and higher levels of Proteobacteria as compared to control mice. Importantly, a similar shift in preponderance of Proteobacteria is known to be associated with increased inflammation [40]. As Gram negative organisms, Proteobacteria contain large amounts of lipopolysaccharide (LPS) molecules within their outer membranes, which are among the most potent inflammatory inducers. Furthermore, Proteobacteria have been associated in other studies with numerous inflammatory conditions, including asthma and atopy [41].

Several murine models of peanut allergy have emerged in recent years [21]. These models differ in their use of mouse strain, route of antigen/adjuvant administration (oral gavage, IP, epicutaneous), use of adjuvants, number of sensitization injections, doses of antigen used, and allergic outcomes assessed [42–47]. Many peanut allergy models use oral gavage to administer the peanut antigen and use cholera toxin as an adjuvant to sensitize mice and favour allergic-like Th2 responses [21, 22]. However, these methods are hampered by the fact that the cholera toxin adjuvant has numerous toxic effects and artificially induces an immune reaction that may not be representative of how peanut allergies naturally occur [21]. Additionally, the gavage route of administration is very stressful for the animal and induces marked stress hormonal responses [48]. Thus, we sought to utilize the naturally occurring pro-inflammatory cytokine, TNF-α, as an adjuvant “immune sensitizer” and avoided the overly stressful gavage in favor of voluntary oral and IP routes of administration.

TNF-α has previously been found to act as an adjuvant capable of augmenting the inflammatory effects of the BSA and house dust antigens [23, 49]. Importantly, TNF-α is endogenously released from mast cell preformed granules upon antigen re-exposure during allergic reactions, thereby contributing to the recruitment of phagocytic and effector immune cells [50, 51]. This may in part explain why TNF-α priming alone (i.e. in the absence of concurrent peanut antigen exposure) elicited some degree of ear swelling in response to the antigen challenge. However, it is important to note that such a response was only evident in mice that previously received the early life antibiotic exposure (Fig. 2), suggesting that TNF-α may either act as an adjuvant/immune sensitizer only within a particular antibiotic-disrupted gut microbiota environment. It is possible that endogenously derived TNF-α plays a role in the development of allergies through inflammatory processes at the epithelial barrier of the gut. Thus, a murine model of peanut allergy employing this protein as an adjuvant may represent a more clinically relevant model that more accurately recapitulates certain elements of the induction phase of human peanut allergies. Indeed, our data suggest that when administered concurrently with peanut butter during the sensitization phase, TNF-α acts as an effective adjuvant.

The localized (ear swelling and itching) allergic phenotypes observed in the present study may have been due to increased baseline inflammatory tone (owing to an elevation of Proteobacteria) or alternatively to a loss of Clostridia bacteria in the gut of the antibiotic-treated mice. Indeed, Clostridiales are among the bacterial orders within the Firmicutes phylum often most affected by vancomycin treatment [52], and they may play a central role in protecting against allergic immune responses under normal conditions [18, 52]. For instance, Stefka et al. [18] previously showed that Clostridia found in the commensal flora play a central role in protecting against peanut allergies by increasing IgA secretion and inducing intestinal epithelial cell and T cell IL-12 secretion to improve the integrity of the intestinal membrane barrier and reduce its permeability to antigens [18]. Moreover, these bacteria can interact with the host to promote the development of intestinal Tregs, which contribute to an anti-inflammatory environment within the gut, [53] and influence the profile of the gut microbiota by inducing the expression of the intestinal antimicrobial peptide, REG3β [18]. It is important to note, however, that the association illustrated in our study does not imply causation and we can only speculate as to possible links between the observed symptoms and the gut microbiota changes. Also, we fully acknowledge that our measures of allergic outcome are somewhat subjective. That said, all measures of swelling and scratching were rigorously calculated by well-trained experimenters blind to the treatment groups.

## Conclusions and caveats

Although we raise the possibility of a link between antibiotic-induced early life disruption to the gut microbiota and later sensitivity to certain immunogenic effects, further studies are required to be able to verify such a link and provide mechanistic details as to the nature of any such relationship. Furthermore, it should be underscored that using different antibiotics might yield different outcomes. Vancomycin is often reserved for serious infections and targets gram positive bacteria such as *Staphylococcus aureus*, which can lead to intestinal inflammation. Other broader spectrum antibiotics would be expected to differentially affect the gut microbiota and hence, potentially impact different allergic outcomes. Moreover, having now shown that broad-spectrum antibiotic exposure induces shifts in the microbiota and affects markers of allergy, future work should also focus on using more narrow-spectrum antibiotics to determine the relative importance of specific populations of bacteria in mediating these immunological effects. To further show a causal role for antibiotic-induced microbial shifts in mediating peanut allergy, fecal transplant experiments aimed at determining the ability of microbial restoration to rescue allergic phenotypes could be useful [54, 55].

The lack of obvious sickness symptoms observed following the peanut protein challenge as would be expected with a systemic or anaphylactic reaction may be related to findings of only elevated circulating IgE levels in sensitized and antibiotic-treated mice. Of course, an important caveat of our study is that we assessed total and not peanut-specific IgE. At the time of this study, we did not have access to this assay and we presently have no biological samples remaining. Thus, we may have missed peanut-specific antibody changes.

Taken together, our findings provide evidence of the utility of TNF-α as an adjuvant in a murine model of peanut allergy and are consistent with the hypothesis that early life exposure to antibiotics alters microbial colonization of the intestinal tract [39] and this was associated with ear swelling and scratching following later intra-dermal peanut antigen challenge. The highly coordinated and intricate relationship that exists between the gut microbiota and host immunity has resulted from millions of years of past evolutionary history; suggesting that some modern lifestyle practices may be disrupting such precisely defined interactions.
